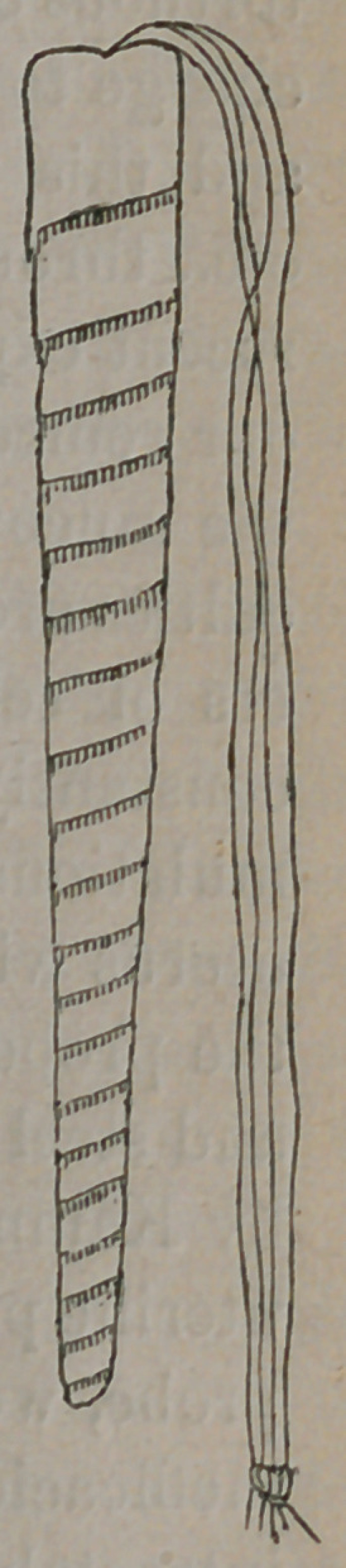# Uterine Cloth Tents

**Published:** 1871-11

**Authors:** V. H. Taliaferro

**Affiliations:** Columbus, Georgia


### UTERINE CLOTH TENTS.

BY V. H. TALIAFERRO, M.D., COLUMBUS, GEORGIA.

I desire to call the attention of the profession to the *uterine cloth tents*, represented in the accompanying wood cuts. Though not perfect, they give a tolerably fair idea of the tent and its manner of preparation.

They should be made of the best quality of bleached domestic, or linen, torn in strips from one-half to one inch in width. These strips should then be compactly rolled between the fore-fingers and thumbs, an obliquity being given the roll from the beginning, so that a firm and delicate point is made and a gradual increase in size obtained as the rolling proceeds, this increase of size being regulated by the degree of obliquity given the cloth strips. To make them perfectly, requires a little practice; but with this, they are easily and quickly made—are firm and pliant, retaining any shape given them. They are designed as a safe, ready and thorough means of intra-uterine medication, dilatation in uterine cervical strictures, and of preparation of the uterus for intra-uterine injections. That they accomplsh these objects with greater safety and thoroughness than any means heretofore employed, I am fully convinced. It will be admitted, by all who are conversant with uterine pathology and therapeutics, that a radical defect has been but too painfully felt in our efforts at intra-uterine medication. The most universally popular plan of treatment to the canal and cavity of the uterus is, perhaps, by means of "the cotton-wrapped probe," as given the profession by Dr. Sims. It certainly has the valuable claim of safety. It is, too, a pleasant and easy mode of treatment; but its results, we are forced to admit, are far from satisfactory. And this is by no means strange, when we reflect that the probe, with its medicated cotton wrapping, can reach the cavity of the uterus only by squeezing through the canal of the cervix. The cotton wrapping is very soft and porous, and requires but little pressure to exhaust it of its fluid contents; and we could not, therefore, reasonably expect that it could be passed through the closed and often tortuous cervical canal, and yet retain a sufficiency of its fluid charge to act therapeutically upon the cavity of the uterus, and this, too, in the presence of mucous, morbid secretions, etc., through which it must pass. We are familiar with the recent experiments of Dr. Nott, of New York, showing how our remedies may become inert by their chemical action upon the mucous and morbid discharges of the uterus. These difficulties are designed to be overcome in the cloth tent. Its powers of self-retention and drainage by exosmosis insures us, constantly, an open canal and a freedom from morbid accumulations; its flexibility enables it to be carried into the uterus with far more ease and less pain to the patient than the probe, with or without the cotton. The fenestrated tubes and steel dilators of Peasley, intra-uterine injections as taught by Kammerer, the sponge tent as utilized by Sims, the intrauterine pessary of Simpson, Lent's syringe and caustic-pointed probe, we must in all candor agree, are fraught with their inefficacies and dangers when brought to bear upon the more intractable diseases of the cavity of the uterus. With the diseases of the cervix, the remedies at our command are usually sufficient; but in chronic mucous and parenchymatous inflammations of the cavity and body of the uterus, we must admit that our treatment, as above enumerated, brings us, constantly, disappointments and perplexities.

Peasley's fenestrated tubes are too expensive and clumsy to be made available for general practice. They require thorough dilatation of the cervical canal before they can be used; and for this purpose Dr. Peasley uses steel dilators, which, in the skillful and experienced hands of Dr. Thomas, proved fatal in at least one case. Dr. Thomas, in speaking of this case, states that on Monday he used three of Peasley's steel dilators, and on Tuesday was summoned to Staten Island, where he says: "I found the lady with violent pelvic peritonitis, of which she died on Sunday evening. I think the mischief was done by the dilatation of the neck of the uterus; yet I am very sure that the dilators were passed with requisite skill, for I spend much of my time in this practice, and this operation is one to which I have constant resort."

With this simple and inexpensive tent, there is no irritation, from unyielding metallic pressure, to threaten us with an acute inflammation in the uterus, or in its surrounding tissues, which may terminate as did the case of Dr. Thomas. Dilatation by steel is rapid, forcible and painful, and must, in consequence, be attended by serious dangers; while with this tent the dilatation is gradual, thorough, more permanent, and unattended by pain or the risk of serious consequences. *The tent both dilates and medicates at the same time.*

Intra-uterine injections, at one time regarded with so much favor, are now resorted to by but few bold admirers of this practice. In urging his objections to this procedure, in the Medical Society of the County of New York, Dr. Thomas says: "Some time since, a gentleman wrote me, from St. Paul, Minnesota, that he had known a case where a small amount of a solution of iodine was injected into the cavity of the uterus, and the woman died suddenly of peritonitis."

This result, in my opinion, might have been avoided by dilatation and *paralysis* of the contractile power of the internal os. The dilatation I consider of far less importance than *complete paralysis of all muscular action* at the internal constriction. This paralytic influence is readily attained by the use of the cloth tents, and I will presently endeavor to show it is requisite in intra-uterine medication, and that with it medicated or non-medicated injections may be made to the cavity of the uterus with the utmost safety. I cannot, therefore, agree with Dr. Thomas, (whom I so much admire, and whose opinions I so much value), that intra-uterine injections "do not constitute an advance in the treatment of uterine diseases." And yet, *without the preparatory measure* here urged, injections into the cavity of the uterus, though powerful for good, are still so encompassed by dangers, that in my opinion, they should be classed with the unjustifiable procedures.

Dr. Henry Miller, of Loüisville, Kentucky, in his retrospect of uterine pathology, in the American Journal of Obstetrics, (August, 1871), states that he has been in the habit, for a number of years, of cauterizing the uterine cavity with strips of lint, passed in with a probe and quickly withdrawn. For some years previous to the adoption of the cloth tent, I had used strips of cloth or linen, saturated with tincture of iodine, solution of carbolic acid, or other substances desired to be used. One end of the strip, folded over the point of a small probe, was passed to the fundus of the uterus; the probe, being withdrawn, was again and again introduced, carrying with it at each time an additional fold of the cloth, until the cavity was completely *packed.* This *packing* was permitted to remain until expelled by the contractions of the uterus, which required, usually, from six to twelve hours. The firmer the packing, the more violent the contractions, and the quicker its expulsion. It was to avoid unnecessary contractile action, and to make the strips self-retaining, that led to the idea of rolling the cloth or linen strip into *a tent,* and thus the better to control, by its size and length, the contractions of the uterus, and to secure its retention for any desirable length of time. Acting upon this idea, the tent was made, and medicated by dropping the fluid to be used upon it until thoroughly saturated. Waiting, then, for a few moments, until the surface had partially dried, a little lard or other simple unguent being applied, it was passed with great ease to the fundus of the uterus.

The following are some of the more important advantages claimed for this tent:

1. Cheapness and simplicity.
2. Its ready manufacture.
3. Great capacity for medication.
4. Its perfect retention of any shape.
5. The readiness and perfect manner with which, by its *joint-like construction*, it adapts itself to any curve or angle of the uterine canal.
6. Facility of introduction into the uterine cavity.
7. Its powers of inducing uterine interstitial stimulation.
8. Its modifying influence by pressure.
9. Its paralyzing influence upon the internal os.
10. Its powers of inducing exosmosis, and consequent drainage from the diseased tissues.
11. It both dilates and medicates at one and the same time.
12. Its efficiency and thoroughness as a means of intra-uterine medication.
13. Its safety.

In using this tent, we have the satisfaction of knowing that the material is exhaustless and always at hand. No instrument maker is to be consulted, no preparatory dilatations to be dreaded and endured, but at once we proceed to the treatment, dilatation following as a consequence, and drainage by exosmosis keeping the uterine cavity freed of all mucus and morbid secretions. The agents with which I am in the habit of medicating the tent are: tincture of iodine and tincture of iodine compound, Lugol's solution of iodine, solution carbolic acid, carbolic acid and iodine in combination, nitrate of silver solution, chromic acid solution, glycerine, chloride of zinc and glycerine, tannin and glycerine, oil turpentine, mucilage, unguents, etc.

We are prone to permit ourselves to become attached to some particular remedy in uterine practice, and confine ourselves almost exclusively to its use, regardless of the pathological condition to be remedied—mucous and parenchymatous inflammations, congestions, indurations, etc., all being alike subjected to the favored remedy. The pathological condition should alone indicate the remedy. If we have parenchymatous inflammation, with interstitial deposits in the body of the uterus, then surely no remedy is so efficient as iodine, or iodine and bromine in combination. These remedies, applied to the uterine cavity by means of the cloth tent, in this character of disease, exercise a restorative influence to be obtained in no other way with which I am acquainted. An ordinary-sized tent will hold from thirty to forty drops of tincture of iodine. This, carried into the cavity of the uterus, is worn by the patient, without special inconvenience, for twenty-four or thirty-six hours, during which time the tent has emptied itself, slowly and gradually diffusing its contents through the diseased tissues of the uterus. At the expiration of some twenty-four hours, the tent will be found freed of its charge of iodine, when it may be replaced by a fresh tent alike medicated. This may ordinarily be repeated from day to day for six or eight days consecutively. I am influenced in its repetition, however, more by the effects produced than by the length of time it has been used. In cases where the uterus is very tender and irritable, a tent every two or three days may be found sufficient.

It will be perceived that by this plan of treatment we give no violent shock to our patient, but, without pain of consequence, or constitutional disturbance, diffuse slowly and gradually from thirty to forty drops of tincture of iodine (or iodine and bromine) through the diseased tissues of the uterus, impressing them very decidedly, and yet imperceptibly, with the peculiar stimulant and alterative action of iodine. Besides the purely medicinal action of the tent, we get its modifying influence by pressure. By carrying the tent to the fundus of the uterus, we obtain a mild and steady contractile action of the uterine muscular fibres, and thus induce a constant and steady *interstitial stimulation.* I invariably direct my patients, that if the uterine pains become so severe as to materially interfere with sleep during the night, to remove it.

To insure a retention of the tent, and for its detergent action, I apply usually a cotton dressing, with glycerine, to the cervix. This dressing is fitted around the cervix in a cup-shape, and carefully kept in its position by means of a probe, as the speculum is withdrawn. With a little care, this dressing is applied so as to fit very nicely around the cervix. The contractions of the uterus, induced by contact of the tent to the fundus of the uterus, I regard with especial favor in parenchymatous diseases, with interstitial deposits. We get in this way, not only a physiological stimulation, but we get, also, *pressure* of the contracting muscular fibres upon the morbid interstitial products. This character of disease Scanzoni admits he has never cured, and Professor Thomas says is the "opprobia of gynæcology."

The amount of serous drainage by exosmosis from the uterine canal and cavity, induced by the medicated cloth tent, is oftentimes astonishing. I have seen the red, tumefied and congested cervix so depleted by a few days' treatment as to present a pale and shriveled appearance. In endometritis, the virtues of the tent are both medicinal and mechanical. In the relief of painful and scanty menstruation, its action is often wonderfully prompt. In illustration, I will give a simple case from my record:

Mrs. B—— came under treatment June 4th, 1871. Aged twenty-seven years; menstruated at fifteen; had one child four years ago; no miscarriage. Hysterical choking; nervous excitability; pain in hips, lower part of the abdomen, vagina and rectum; pain upon defecation in rectum and vagina. Weighs now one hundred and fifteen pounds; formerly, one hundred and forty. Irritable bladder, with too frequent micturition. Menstruation every four weeks; *scant and exceedingly painful*, with greatly increased constitutional disturbance, aggravated hysteria, etc. Profuse white leucorrhœa constant for the past twelve months. By physical examination and conjoined manipulation, we find a tender retroverted uterus. By the sound, introduced without obstruction at the internal os, we find pain and three and a half inches depth of cavity. The diagnosis in this case is chronic corporeal metritis with endometritis, caused most probably by some accident following child-birth. The dysmenorrhœa has been so distressing only for the past twelve months. The latter part of July, the second menstruation was *free and painless.* From this time to the date of the patient's discharge, October 15th, there was no pain attending the menstrual flow. Up to this date, November 4th, she has been free of her dysmenorrhœa, hysteria, leucorrhœa, pains, etc.

This patient was discharged before she was believed to be entirely well, and sent to the country with a Cutter's retroversion pessary, for the recruit of her general health, which was suffering from constant confinement and treatment. I saw her a few days since, greatly improved, and menstruating without pain. If she gets along well, I shall not examine her under two or three months, to determine if she requires further treatment.

The *constant contact and pressure* of the cloth tent upon the muscular fibres of the internal os-uteri *completely overcomes and paralyzes its contractile power.* We are thus enabled, after one or two tents, to *thoroughly wash out the uterine cavity*, preparatory to a continuance of the treatment by tents or by injection. If the tent has retained its position perfectly for twenty-four hours, there need be no hesitation in washing out the uterus with a proper syringe. When warm water was not convenient, I have very often used cold water, and without causing the least trouble, when the internal os has not been paralyzed in the way we have described, or by some similar process. The first effect of a uterine contraction is its complete closure. I am convinced now, by numerous experiments, that the closure of the internal os is the initiative of a general contraction by the uterus, and that before the impression of the contractile action has been elsewhere felt, it has been completely closed, becoming more and more unyielding as the contractions increase in intensity. Hence it is, that however much may be the dilatation of the cervical canal and internal os as the results of inflammatory action, intra-uterine injections are none the less hazardous. These being facts, *the cause* of uterine colic, collapse and death from injections into the cavity of the uterus, is more clear. A confined fluid within the cavity of the uterus, subjected to the immense power of its violent contractions, finds its only outlet through the Fallopian tubes and into the peritoneal cavity. When we remember the great *contractile power* residing in the uterus, we can feel no surprise that a fluid confined within its cavity, exciting its intensest action, should be forced through the Fallopian tubes and carried into the peritoneal cavity. By the application of this powerful pressure, it is doubtless forced through the Fallopian tubes *in jets or streams* of very considerable force, simulating to some extent the action of a Davidson syringe in operation, with a strong hand applied to its bulb. The uterine openings of the Fallopian tubes, in health, are very minute, but we have reasons for believing that they are often much dilated by disease, which has extended to them from the cavity of the uterus. Their size, however, whether minute or large, would influence *only the size* of the stream admitted through them.

We are told by Dr. Kammerer and others that intra-uterine injections may be made comparatively safe by first dilating the cervical canal with sponge or laminaria tents. As already stated, it is not, in my opinion, the dilatation simply which is needed, but *paralysis.* With this complete, it matters not if the opening into the uterine cavity be small, so that it admits freely the tube or pipe of the syringe, and is not completely filled up by it. The paralysis of the internal os being perfect, injections into the cavity of the uterus may be made with the utmost freedom and safety, so far as concerns any immediate results, as uterine colic, collapse, etc. Caustic injections may be so concentrated as to cause serious after-consequences; but these are far less hazardous, with a paralyzed internal os, than the injection of the blandest substance without the observance of this precaution. When the cloth tent is used simply for the purpose of dilatation and paralysis of the internal os, it is best saturated with glycerine—or, if there is considerable irritability, a little opium or extract of hyoscyamus may be added. Thus prepared, the tent is not only harmless, but relieves congestion and irritability of the mucous surface by its depletory action. If any considerable amount of dilatation is desired, the tents may be increased in size from day to day. This gradual and painless dilatation is, I am very sure, unattended by the dangers of rapid and forcible dilatation, as obtained by sponge, steel dilators, etc., now in general use for that purpose.

The cloth tent, when well prepared, is well-introduced into the cavity of the uterus with greater ease than the sound, bougie, or indeed anything else I have ever attempted. This is apparent from its peculiar construction, its firmness and pliability. We see that it is *jointed*—every separate roll of the cloth strip constituting a joint—thus giving an easy mobility, in every direction, to all its parts. This facility of introduction enables us to use it, as I often do, as a means of diagnosis in cases of flexions, versions, tortuous uterine canals, etc., in which difficulty is found in passing the probe. It adapts itself, with great readiness, to any curve or angle of the uterine canal, and upon withdrawal, gives the direction we are to take in the introduction of the probe. It retains any shape of curve given it, and hence gives us the exact mould, so far as shape is concerned, of the uterine canal. When delicately and firmly made, it will easily follow the angle of a flexion, so abrupt as utterly to preclude the passage of a sound. I have at this time a case of chronic metritis, with a decided anti-flexion, in which I was greatly assisted in diagnosis by the tent, it being the only thing I was able to pass through the tortuous and abruptly-curved uterine canal. This patient (a virgin) contracted her disease from suppressed menstruation in girlhood. The uterus measures three and three-eighth inches in depth. In this case, we would wish to fulfill three important indications, viz: First, direct medication; second, interstitial stimulation; third, absorption. The medicated cloth tent, in my opinion, will more efficiently accomplish these objects than any means at our command. The tent should be sufficiently long to reach the fundus, and medicated with iodine and bromine. A little simple cerate or suet then thoroughly applied to its surface, and it is ready for use. With uterine dressing forceps or tent carrier, it is carried through the uterine canal to the fundus of the uterus, where it is permitted to remain twenty-four hours. During this time, it has discharged its medicinal agents directly into the diseased tissues. Its pressure in the cavity and at the fundus of the uterus excites a mild contractile action, giving us a very decided and thorough *interstitial stimulant.* This, together with the alterative and stimulating influence of the iodine and bromine, must induce, in the diseased tissues, the utmost absorbent action of which they are capable. This treatment may be continued for a number of days consecutively, or until the patient gives some evidence of constitutional disturbance, indicating its cessation.

Sub-involution of the uterus—first described, I believe, by Professor Simpson, of Edinburgh—is the arrest, following parturition, of "the fatty metamorphosis of the uterine muscular fibres," leaving "a hypertrophy of the uterus, which is pathological in its permanency, but which results from a hypertrophy purely physiological in its results." Professor Simpson, in the treatment of this condition, says:

"If, however, by any means, you can induce the uterus for a time to take on an action of increased growth, you may confidently hope that this temporary hypertrophy will be followed by a process of absorption, which will go on, perhaps uninterruptedly, until the organ is reduced to its normal dimensions."

The tendency of the uterus to enlarge is the same, whether from the artificial stimulation of a foreign substance or the physiological stimulation of an ovum. Professor Simpson has taken advantage of this tendency, and in the treatment of sub-involution, introduces into the cavity of the uterus small bits of sponge, or his metallic intra-uterine pessary. The dangers which beset these agents are too well known to the profession to require mention here. The cloth tent, in my opinion, certainly recommends itself as the agent best calculated to safely and efficiently fulfill the indications pointed out by Professor Simpson. It will accomplish the purposes designed by the sponge, and without its evil effects; it will fulfill the designs of the intra-uterine pessary, without its hazardous consequences.

A case of sub-involution under my care was subjected for eighteen months to sponge tents, caustics, intra-uterine injections, blisters, bromine, iodine, mercury, etc., without appreciable reduction in the size of the uterus, though the menorrhagia, which had been her chief complaint, was entirely relieved and her general health much improved. Becoming convinced of the utter inefficiency of these remedies to reduce the enlarged organ, I had manufactured an *intra-uterine pessary*, consisting of a very small elastic *india-rubber sac*, attached to a hard rubber tubular stem, some eight inches in length, and about the size of a No. 3 bougie. The uterine extremity of the stem is given any desired curve required by the position of the uterus, while the vulva extremity is curved backward over the cervix, after the plan of Cutter. To the curved vulva extremity is attached the elastic india rubber tubing, and this secured to a belt around the waist. By this simple arrangement, the instrument is very securely fixed in its position. The tubular stem, being small, has considerable elasticity. The instrument, being thus arranged, is passed into the cavity of the uterus as an ordinary probe. By means of a syringe, the little sac is now distended with water, when it becomes immovably fixed. In this patient, the instrument was used for six days consecutively, the sac being daily emptied and refilled. The cavity of the uterus was very rapidly dilated, so that on the sixth day it contained more than a half ounce of water. The contractions of the uterus, induced by this distension of its cavity, were persistent and violent, and although I instructed her to empty the sac at any time the pains became very severe, she persisted in wearing it.

On the sixth day, the sac was distended to its very fullest capacity. I now directed my patient positively to empty the sac, should the pains keep up with violence. This was in the morning. In the latter part of the night, I was sent for. When I arrived, I found my patient just over a severe chill. Thinking she would go through with the pains as she had previously done, she failed to empty the instrument until the chill came on. This patient went through a severe attack of peri-uterine cellulitis, suppuration and discharge by the rectum. I had not then used the instrument sufficiently to know how to graduate the distension. In a future communication, I will give the history of this instrument more in detail, together with its results in flexions, versions, etc.

After the recovery of my patient from her attack of cellulitis, I found the uterus a little reduced in size, and not so hard. I now commenced her treatment by the *cloth tent*, which, in three months, was suspended to give her rest and a trip to the country. Upon her return, after an absence of eight or ten weeks, she was feeling quite stout and well, with an absence of leucorrhœa and all uterine trouble. Upon an examination of the uterus, it was found greatly improved in appearance, very much softer, and a half inch reduction in depth of character. She was discharged, with directions to return should any untoward symptom present itself. Some twelve or fourteen months have now elapsed, and my patient has had no return of her old troubles. She leads an active life, and has enjoyed most perfect health.

The character of disease to be treated should be our guide to the character of medication for our tent. In very irritable and tender conditions of the uterus, the tent should at first be medicated with the simplest unguents, combined with a little opium, hyoscyamus, or belladonna, or with glycerine, elm or flax-seed mucilage. The tent thus prepared, warmed as hot as can be comfortably borne, and introduced into the uterine canal, affords great relief in irritable and painful conditions.

I beg the liberty of insisting, in commencing the treatment of uterine diseases with this tent, that it be medicated with the mildest substances, until the susceptibilities and idiosyncracies of the patient have been carefully ascertained. Until I became, myself, sufficiently guarded in this particular, I came near causing the death of two patients with chromic acid, and in neither of whom was the remedy carried as far as the internal os. The effects upon each of these patients were intense nausea and vomiting, great prostration, cold and clammy surface, and feeble circulation. I feel quite sure, that had I carried the remedy into the cavity of the uterus in these two cases, it would have caused the death of them both. I have often cauterized the uterine cavity with chromic acid, for it is a remedy I very much admire, and only in these instances have I witnessed any unpleasant effects. These cases, however, have taught me to use it with exceeding caution, and to carry it into the cavity of the uterus with fear and trembling; and I now never do so until I have first tested its effects upon the external os, and then upon the cervical canal. I cannot agree with Dr. Miller, that the *cavity* of the uterus is as tolerant of remedies as the cervix and its canal. In chronic metritis and endometritis, where a very decided impression is desired, I often terminate each successive treatment by injecting into the cavity of the uterus tincture of iodine compound, and by a thorough application of chromic acid by means of the tent. When I have reason to fear the effects of the chromic acid, I use, instead, carbolic acid or the acid nitrate of mercury. When these caustic applications are made to follow the tent, the intervals in the treatment must, of course, be lengthened.

In conclusion, I must apoligze for the hasty and desultory manner in which these thoughts have been thrown together.

## Figures and Tables

**Figure f1:**
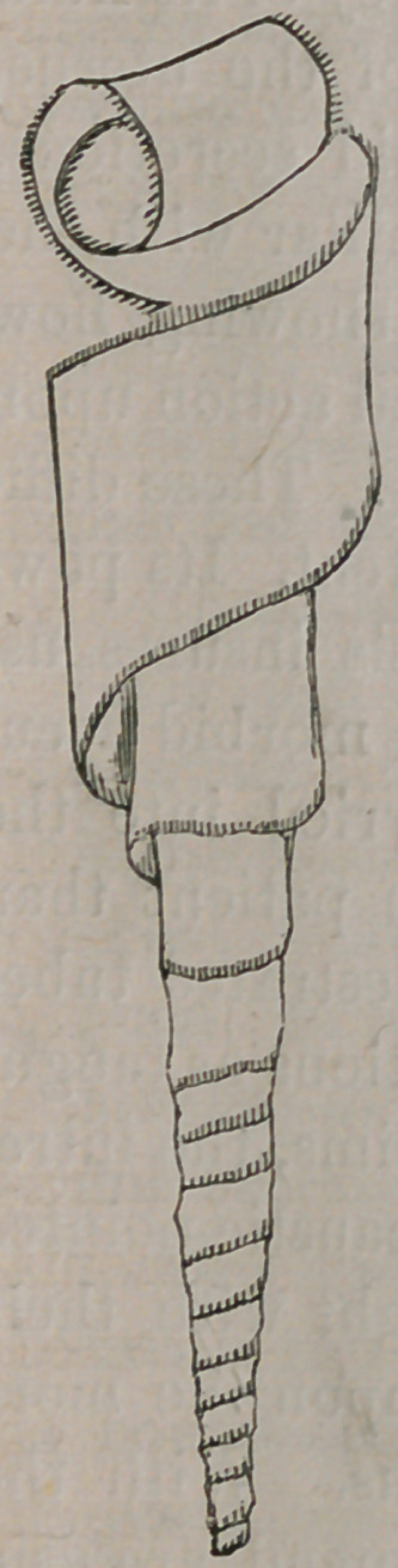


**Figure f2:**